# A comparative study of effects of microwave-assisted deep eutectic solvents pretreatment of rice straw on cellulose enrichment and enzymatic digestibility

**DOI:** 10.1007/s10570-025-06834-y

**Published:** 2025-11-06

**Authors:** Longinus Ifeanyi Igbojionu, Yujie Mao, Eleanor Binner, Alfred Fernandez-Castane

**Affiliations:** 1 Energy and Bioproducts Research Institute (EBRI), Aston University, Aston Triangle, Birmingham, B4 7ET UK; 2 Aston Institute for Membrane Excellence (AIME), Aston University, Aston Triangle, Birmingham, B4 7ET UK; 3 Department of Chemical and Environmental Engineering, Faculty of Engineering, University of Nottingham, University Park, Nottingham, NG7 2RD UK

**Keywords:** Deep eutectic solvents, Microwave-assisted pretreatment, Cellulose enrichment, Enzymatic digestibility, Rice straw

## Abstract

Lignocellulosic biomass (LCB), such as agricultural residue rich in cellulose and hemicellulose, can serve as a feedstock for bioethanol production due to its environmental benefits. Deep eutectic solvents (DES) are low-cost and greener solvents for LCB pretreatment. The present study investigated the efficiency of two DES types for rice straw pretreatment under microwave conditions: choline chloride:glycerol (ChCl:Gly) and choline chloride:formic acid (ChCl:FA). DES pretreatment was performed under microwave conditions (100–140 °C and 5–15 min) at 10% solids loading and constant power 200W. After pretreatment, the samples were characterized, and enzymatic digestibility was investigated at 50 °C for 72 h using enzyme loading of 6 filter paper unit g^−1^ cellulose and 10% solids loading. Untreated rice straw consists of cellulose (41.8%), hemicellulose (24.9%), lignin (17.0%) and ash (15.0%). The cellulose content increased to 59.8% and 59.2% after ChCl:Gly and ChCl:FA pretreatment, respectively, while hemicellulose decreased to 15.6% and 10.1% after ChCl:Gly and ChCl:FA pretreatment, respectively. Lignin content decreased to 8.2% after ChCl:Gly pretreatment compared to 9.6% after ChCl:FA pretreatment. Ash content (20.4%) obtained after ChCl:FA pretreatment was higher than 14.0% obtained after ChCl:Gly pretreatment. The cellulosic and hemicellulose fractions from ChCl:Gly pretreatment were effectively hydrolyzed with glucose and xylose yield of 86.0% and 68.4%, respectively, compared to glucose and xylose yield of 58.2% and 62.8%, after ChCl:FA pretreatment. ChCl:Gly pretreatment enriched cellulosic content and preserved hemicellulose fraction, achieving a higher yield of fermentable sugars than ChCl:FA pretreatment; hence, it can potentially support a biorefinery.

## Introduction

Lignocellulosic biomass (LCB), including agricultural residues, is rich in cellulose and hemicellulose that can serve as a feedstock for bioethanol production, mainly due to its environmental benefits (Broda et al. 2022). Bioethanol is produced primarily from sugar and starch-rich crops like sugarcane, corn, cassava, and potatoes, which presents significant drawbacks mainly due to competition for agricultural land and resources (Mahapatra et al. 2021). However, agricultural residues such as rice straw are typically underutilized, and converting them into bioethanol helps to reduce waste, minimize landfilling, and lower greenhouse gas emissions (Kumar et al. 2020; Sharma et al. 2020; Singh et al. 2017). China and India each produce ~ 207 million tons of rice straw annually, accounting for about 52% of global paddy and rice straw (Alengebawy et al. 2023). In addition to addressing food security and environmental concerns, this approach could create new economic opportunities, particularly in rural areas where large quantities of agricultural residues occur (Porichha et al. 2021). Rice straw accounts for an estimated 1.1 billion tons from over 6 billion tons of agricultural residues generated globally annually, making it a key resource for potential biofuel production (Becerra-Pérez et al. 2022; Cherubin et al. 2018).

Rice straw is composed of cellulose (35–50%), lignin (10–25%), and hemicellulose (20–35%), with lignin being tightly bound to the cellulose and hemicellulose, resulting in a highly rigid structure (Kumari and Singh 2022; Chen et al. 2020). The recalcitrance of rice straw, while providing structural integrity and protection to the plant, poses a significant challenge to its utilization as a feedstock in biofuel production. The rigid structure limits the accessibility to cellulose and hemicellulose, which is essential for bioethanol production (Hendriks and Zeeman 2009). Therefore, optimizing the pretreatment process is critical for improving the efficiency and yield of subsequent enzymatic hydrolysis and fermentation steps (Igbojionu and Laluce 2023; Mankar et al. 2021; Cai et al. 2017).

Currently, pretreatment remains a significant bottleneck in the industrial-scale production of bioethanol from lignocellulosic biomass, accounting for approximately 40% of the total production cost (Njoku et al. 2012; Broda et al. 2022). The physical, chemical, physicochemical, and biological pretreatment methods have been applied to LCB (Baruah et al. 2018; Hierro-Iglesias et al. 2024). Deep eutectic solvents (DESs) have recently attracted attention as a promising alternative for biomass pretreatment due to low cost ( < $2/kg), ease of synthesis, reusability, and superior dissolving capability (Maniam and Paul 2021; Chen and Mu 2019; Grillo et al. 2021; Monroy et al. 2024). Typically, DESs consist of a hydrogen bond acceptor, often choline chloride (ChCl), and a hydrogen bond donor, which could be compounds like oxalic acid, urea, or polyols (Abbott et al. 2004; Smith et al. 2014). One of the primary scientific challenges in utilizing acid-based DES such as choline chloride:formic acid (ChCl:FA) for biomass pretreatment is that, while these solvents can achieve high delignification efficiency, they also degrade polysaccharide fractions, thereby diminishing the valorization potential of the residual biomass (Oladzad et al. 2024; Jönsson and Martín 2016).

In response to these issues, researchers have turned to neutral polyol-based DESs, such as ChCl:glycerol (ChCl:Gly), for the pretreatment of LCB. These neutral DESs have shown promise in reducing polysaccharide degradation (Igbojionu et al. 2025; Wang et al. 2022, 2020; Hassan and Mutelet 2022), but the problem of high viscosity and low thermal conductivity persist with ChCl:Gly, limiting its effectiveness during conventional heating methods (e.g., autoclave and oil bath). This problem is solved by applying microwave-assisted heating to accelerate the delignification process and enhance enzymatic digestibility (Mao et al. 2023; Galan et al. 2017; Chen et al. 2022; Kostas et al. 2017). Microwave-assisted ChCl:FA pretreatment can boost lignin removal by 24–90% and 60–76% with ChCl:Gly + catalyst compared to ~ 24% lignin removal using conventional heating at 130–140 C (Mao et al. 2023; Lozano Pérez et al. 2024; Zhang et al. 2025a, b). Thus, the use of microwave enhances extraction efficiency, shorten processing time, and attain superior lignin extracts (Mankar et al. 2021). Additionally, microwave-assisted treatment enhances biomass porosity and lowers crystallinity, increasing enzyme accessibility and high sugar yield (Kumar et al. 2019).

Previous studies of DES pretreatment focused mainly on applying individual DES for the pretreatment of rice straw (Hossain et al. 2021; Sawhney et al. 2023; Kumar et al. 2019). Studies directly comparing the effectiveness of ChCl:Gly and ChCl:FA pretreatment under microwave conditions are still missing in the literature. Thus, a comparative study of the two types of DES (ChCl:Gly and ChCl:FA) under microwave conditions could provide insight into the merits and demerits of each DES, while allowing process optimization. To this end, the present study aimed to compare the efficiencies of ChCl:Gly and ChCl:FA pretreatment of rice straw under different microwave conditions. Secondly, the effects of DES pretreatment on delignification, composition, cellulose crystallinity, structure, thermal properties and cellulose digestibility were assessed and compared.

## Materials and methods

### Materials

Rice straw was obtained from a rice farm in Akure, Ondo State, Nigeria (7.250771, 5.210266). The straw was sun-dried to a 10% moisture content and stored in an airtight plastic bag at room temperature. Straw was ground using a knife mill (CM4000, LAARMANN, Roermond, Netherlands) and sieved by passing through a set of sieves (mesh 32 and 35) to obtain a particle size of ≤ 0.5 mm and was subsequently stored in an airtight container and kept at room temperature before use. All chemical reagents used in this study were purchased from Fisher Scientific Ltd (Leicestershire, UK). The Cellic® CTec3 HS was a kind donation by Novozymes A/S (Bagsværd, Denmark).

### Preparation of DES

Deep eutectic solvents consisting of ChCl:Gly and ChCl:FA mixtures were prepared at a molar ratio 1:2 (Zhang et al. 2022). The mixtures were heated to 80 °C with constant stirring (400 rpm) until the mixture became transparent and homogeneous. ChCl:Gly and ChCl:FA mixtures were later transferred into a vacuum drying oven at 50 °C for 24 h to remove the water present. Afterwards, ChCl:Gly and ChCl:FA mixtures were removed, allowed to cool down and stored at room temperature before use.

### Pretreatment

The microwave-assisted DES pretreatment was carried out using a Monowave 200 microwave (Anton Paar, Graz, AT) at 2.45 GHz. 1 g of straw was mixed with 10 g of DES in the Monowave reaction vial. A maximum incidence power of 200 W was applied with stirring at 600 rpm, and the holding temperature and reaction time varied from 100–140 °C to 5–15 min, respectively as described by Mao et al. (2023). After each reaction, compressed air was used to cool down the sample to 70 °C before the vial could be removed from the Monowave chamber. 10 mL of 30% ethanol was used to transfer the sample from a microwave vial into a centrifuge tube before centrifuging at 3900 rpm for 15 min (Heraeus Multifuge X1R, Thermo Scientific, Massachusetts, USA). The supernatant containing soluble lignin was decanted. The solid residue was first washed with another 10 mL of 30% ethanol, followed by centrifugation (Mao et al. 2023). 20 mL of deionized water was used to wash the sample twice and filtered using Whatman filter paper under vacuum. Solid residue recovered was dried at 50 °C for 30 h, cooled down, and stored in an airtight container.

The solid residues after pretreatment (expressed as wt% on dry biomass) were calculated using the weight (w) difference before and after pretreatment according to Oladzad et al. (2024) as shown in Eq. 1 (Eq. 1).

1 $$Solid\; recovery \left( \% \right) = \frac{{W_{initial \;biomass} - W_{final \;biomass} }}{{W_{initial \;biomass} }} \times 100$$

The efficiency of lignin removal or the delignification efficiency was calculated by accounting for the weight difference between lignin present in the untreated straw and pretreated sample as described by Poy et al. (2023) using Eq. 2.

2 $${\text{Delignification }}\;{\text{efficiency }}\left( {\text{\% }} \right) = { }\frac{{{\text{\% of lignin }}\left( {{\text{raw}}\;{\text{ sample}}} \right) - {\text{ \% of lignin }}\left( {{\text{pretreated }}\;{\text{sample}}} \right)}}{{{\text{\% of}}\;{\text{ lignin }}\left( {{\text{raw }}\;{\text{sample}}} \right)}} \times { 1}00$$

### Composition analysis

The composition (cellulose, hemicellulose, lignin and ash) of the untreated and pretreated samples was determined using a method of National Renewable Energy Laboratory (Sluiter et al. 2012). 300 mg of sample was placed in a glass tube, and 3 mL of 72% H_2_SO_4_ was added. The sample was gently mixed, and the tube was incubated in a water bath at 30 °C for 1 h with stirring at 5–10 min intervals using a glass rod. Afterwards, the sample was diluted to 4% H_2_SO_4_ with 84 mL of deionized water in a 100 mL screw-capped pressure tube. Sugar recovery standards were prepared by weighing 150 mg of glucose, xylose, arabinose, galactose, and mannose. 348 µL 72% H_2_SO_4_ was added, and the tube was vortexed. The tube was capped and placed in an autoclave (Heraeus Multifuge X1R, Thermo Scientific, Massachusetts, USA) and heated at 121 °C for 1 h. The sample was removed from the autoclave after cooling down to 50 °C, and the sample was allowed to cool down to a room temperature before it was filtered using a dry and pre-weighed crucible under a vacuum. The crucible was transferred into an oven at 105 °C and allowed to dry to a constant weight to determine the acid-insoluble residues (AIR). The crucible was transferred into a muffle furnace (Lenton, Lenton Thermal Designs, Derbyshire, UK) and heated at 600 °C for 8 h to determine the ash content.

Before the neutralization of the filtrate, a portion of it was used to measure the acid-soluble lignin via a spectrophotometer at 240 nm. Neutralization was carried out using CaCO_3_, and the sample was neutralized to a pH of around 5 by following a standard protocol (Sluiter et al. 2012). The neutralized acid hydrolysate was filtered through a 0.22 µm filter before it was used for sugar analysis.

Sugar analysis was carried out using a High-Performance Liquid Chromatography (HPLC) (1260 Infinity) equipped with a Hiplex H column (Agilent) and connected to a Refractive Index Detector. The sugar recovery standard consisted of glucose, xylose, arabinose, galactose, and mannose. The HPLC conditions were the following: 5 mM H_2_SO_4_ was the Mobile phase with a flow rate of 0.6 mL min-1, 25 °C was the column temperature, and the detector temperature was 35 °C.

### X-ray diffraction analysis

A sample of uniform particle size (≤ 0.5 mm) was carefully placed on the sample holder and gently pressed down with a flat glass slide. The holder was placed gently on the sample stage, and scanning was applied ranging from 10 to 80° (2θ) at 6°/min. The X-ray diffractometer (D8 Advance instrument, Bruker, Germany) was operated at 40 kV of voltage and a current of 40 mA. The crystallinity index (CrI) was calculated by employing the method described by Segal et al. (1959).

3 $${\text{CrI }}\left( \% \right) \, = (I_{200} - I_{am} )/I_{200} \times 100$$

where *I*_200_ and *I*_am_ represent the diffraction intensity at 22° and 18° respectively.

### Attenuated total reflection (ATR)-Fourier-transform infrared (FTIR) analysis

ATR-FTIR spectroscopy is mainly used to detect different functional groups present in biomass (Awoyale and Lokhat 2021). ATR-FTIR instrument (Nicolet iS50, Thermo Scientific, Massachusetts, USA) was used for the analysis. ATR-FTIR spectra of the blank were first recorded over a wavenumber ranging from 400 to 4000 cm^−1^. Afterwards, a sample of uniform particle size (≤ 0.5 mm) was placed on the sample holder, and ATR-FTIR spectra were recorded using the same wave number as the blank.

### Scanning electron microscopy

Scanning electron microscopy (SEM) scans a sample to produce a magnified image for surface visualisation. The SEM instrument (JEOL JSM 7800F PRIME, JEOL Ltd, Hertfordshire, UK) with a magnification in the range of 20 -100,000 × was used. A sample of uniform particle size was placed on a stub and adhered with carbon tape. The stub was inserted into the sample stage and tightened into place. The image of the sample was captured at a magnification of 1000×.

### Thermogravimetric analysis

TGA analysis was carried out using the Thermogravimetric Analyzer instrument (TGA/DSC 2, Mettler-Toledo Ltd, Leicester, UK). Around 3 mg of the sample was weighed into a crucible. An empty crucible (blank) and a crucible containing the sample were loaded into the TGA autosampler. The heating was performed using nitrogen as the carrier gas, and the flow rate was set at 70 mL min^−1^. Heating temperature was set in the range of 40 to 400 °C with a rate of 10 °C min^−1^. Thermogravimetric and differential thermogravimetric curves were plotted after subtracting the blank from the test sample using OriginPro software (OriginLab Corporation, Northampton, USA).

### Enzymatic hydrolysis

Enzymatic hydrolysis was performed using Cellic® CTec3 HS with cellulase and ß-glucosidase activities. The enzyme activity of 106 filter paper unit (FPU) mL^−1^ was determined using the method described by Ghose (1987). 500 mg of sample (10% w/v) was loaded into a 15 mL screw-capped glass tube containing 5 mL sodium citrate buffer (50 mM, pH 4.8) and enzyme loading of 6 FPU g^−1^ cellulose. Sodium azide (0.5 g/L) was added to the mixture to prevent contamination and microbial growth. Afterwards, the tube was vortexed to allow proper mixing of the enzyme with the sample (substrate). The hydrolysis was conducted in a shaking incubator (Incu-Shake MAXI, SciQuip Ltd, Rotherham, UK) at 50 °C for 72 h and shaking at 150 rpm. After 72 h, the sample was deactivated by a heating block (100 °C) for 10 min. Later, the sample was removed and allowed to cool down to room temperature, and the supernatant was collected for sugar analysis using HPLC under the conditions described before.

The yield of glucose and xylose was calculated using the Eq. 4 below.

4 $${\text{Glucose}}/{\text{Xylose yield }}\left( {{\text{\% }}} \right) = \frac{{{\text{Total}}\;{\text{amount}}\;{\text{of}}\;{\text{glucose}}/{\text{xylose}}\;{\text{in}}\;{\text{the}}\;{\text{enzymatic}}\;{\text{hydrolysate }}\left( {\text{g}} \right)}}{{{\text{Initial}}\;{\text{amount}}\;{\text{of}}\;{\text{glucan}}/{\text{xylan}}\;{\text{in}}\;{\text{the}}\;{\text{substrate }}\left( {\text{g}} \right)}}{ } \times 100$$

### Statistical analyses

Plotting of figures were performed using Origin Pro V2024. All experiments were conducted at least in duplicate, and the results were presented as mean ± standard error of the mean. Statistical analysis of data was performed using SPSS Statistics (IBM SPSS Statistics, Version 29.0., USA). Data were subjected to a one-way ANOVA with the Duncan test and the probability value of *p* < 0.05 was considered significant.

## Results and discussion

### Effect on yield of solids residue

**Fig. 1:**
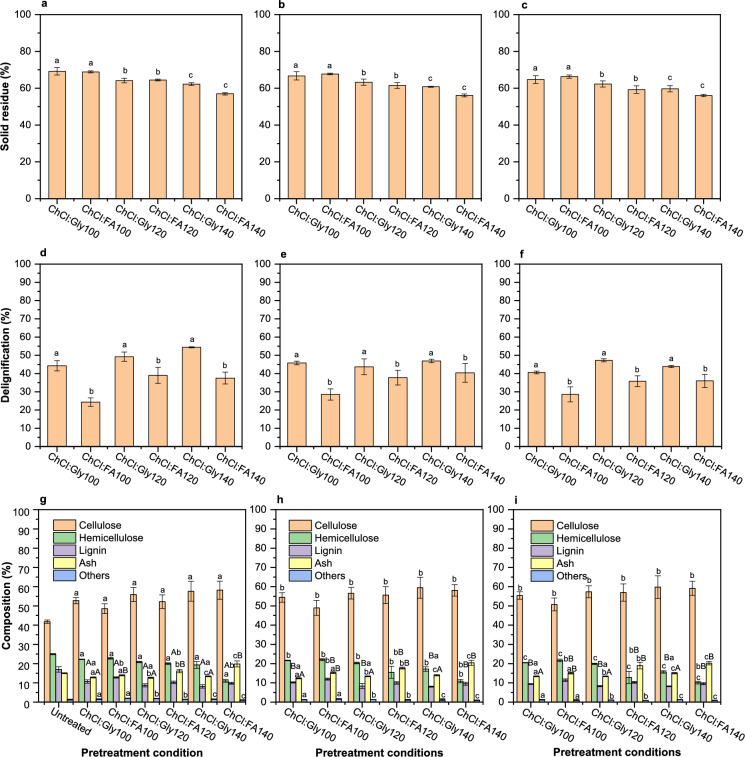
Effect of ChCl:Gly and ChCl:FA pretreatment on biomass: **a–c** Yield of solid residue at 5 min, 10 min, and 15 min, respectively; **d–f** Delignification at 5 min, 10 min, and 15 min, respectively; **g–i** Biomass composition at 5 min, 10 min, and 15 min, respectively; Average values are presented, and the error bars correspond to standard deviation; Different letters in the same data figure indicate the significant difference (*p* < 0.05) disrupting the lignin and carbohydrate complex and subsequent lignin removal (Chen et al. 2024). The greater impact of rising temperatures on ChCl:FA pretreatment compared to ChCl:Gly pretreatment is due to the lower thermal conductivity of ChCl:FA under microwave irradiation treatment. Thus, the differences between ChCl:Gly and ChCl:FA pretreatment on the yield of solids residue may be due to hemicellulose degradation at higher temperatures under acidic ChCl:FA compared to the neutral ChCl:Gly, which tends to favour lignin removal selectively (Sha et al. 2024; Tian et al. 2020)

Figure 1a–c shows the effect of DES pretreatment on the yield of solid residue. At 5 min (Fig. 1a), temperature increases decreased the yield of solid residue to 69.2% and 68.9% after ChCl:Gly and ChCl:FA pretreatment respectively at 100 °C. As the temperature increased to 120 °C, solid residue decreased to 64.2% and 64.5% after ChCl:Gly and ChCl:FA pretreatment, respectively. Further increases in temperature to 140 °C resulted in 62.3% and 57.0% of solid residue after ChCl:Gly and ChCl:FA pretreatment, respectively. In Fig. 1b, solid residue decreased to 66.7% and 67.8% after ChCl:Gly and ChCl:FA pretreatment at 100 °C, respectively. Increasing the temperature to 120 °C resulted in 63.3% and 61.5% of solid residues after ChCl:Gly and ChCl:FA pretreatment, respectively. At a higher temperature of 140 °C, solid residue decreased to 60.8% and 56.1% after ChCl:Gly and ChCl:FA pretreatment, respectively. Similarly, as shown in Fig. 1c, ChCl:Gly and ChCl:FA pretreatment at 100 °C, 15 min, resulted in 64.7% and 66.4% of solid residues, respectively. Increases in temperature to 120 °C, resulted in 62.3% and 59.7% of solid residue after ChCl:Gly and ChCl:FA pretreatment, respectively. The yield of solid residue further decreased with increases in temperature to 140 °C, resulting in 59.7% and 56.1% of solid residue after ChCl:Gly and ChCl:FA pretreatment, respectively. Hossain et al. (2021) reported decreases in the yield of solid residue ranging from 78 to 67% after ChCl:Gly pretreatment was applied to rice straw at 150 °C for 3–24 h. Other authors reported 53% yield of solid residue from sugarcane bagasse after ChCl:FA pretreatment at 110 °C for 60 min (Ling et al. 2021).

The similarity of temperature and time effects was observed in the yield of solid residue in both DES, mainly at a lower temperature (100 °C). However, the impact of temperature increases appeared to be more pronounced on the yield of solid residue compared to residence time. Lignin solubilization increases at higher temperatures,

### Effect on delignification

The effect of DES pretreatment on delignification is shown in Fig. 1d–f. In Fig. 1d, 24.4% delignification was obtained after ChCl:FA pretreatment at 100 °C and later increased to 39.0% at 120 °C, while after ChCl:Gly pretreatment, delignification rose from 44.3% (100 °C) to 54.4% (140 °C). Similarly, as shown in Fig. 1e, delignification increased from 45.8% (100 °C) to 46.9% (140 °C) after ChCl:Gly pretreatment, compared to delignification of 28.5% (100 °C) to 40.4% (140 °C). In Fig. 1f, delignification increased from 40.6% (100 °C) to 43.8% (140 °C) after ChCl:Gly pretreatment, while delignification of 28.6% and 36.0% were obtained after ChCl:FA pretreatment at 100 °C and 140 °C, respectively. However, the observed differences in lignin removal between ChCl:Gly and ChCl:FA may be due to their dielectric properties. The dielectric properties of DES significantly influence their heating rates under microwave conditions, particularly favouring faster heating in acidic systems (Mao et al. 2023). Isci et al. (2020) demonstrated that microwave pretreatment with acidic DES like ChCl:FA can accelerate lignin removal and increase lignin condensation risk, especially under rapid heating conditions. Other authors noted that strong acidic environments and high temperatures can trigger lignin re-polymerization and condensation reactions during biomass pretreatment (Tan et al. 2025). At high temperatures the breakdown of ester and ether linkages within lignin accelerates, allowing better dissolution in the DES solution (Zou et al. 2024). In this present study the optimal delignification occurred at 140 °C for both ChCl:Gly and ChCl:FA, facilitating high rate of lignin solubilisation and removal from rice straw.

However, delignification decreased after optimal delignification was reached, suggesting lignin repolymerization and formation of pseudo-lignin (Anuchi et al. 2022; Chambon et al. 2021; He et al. 2020). The highest delignification of 54.4% was obtained using ChCl:Gly compared to 40.4% obtained with ChCl:FA, indicating the high efficiency of ChCl:Gly pretreatment under microwave conditions. Other authors reported the delignification efficiency of 52% from rice after ChCl:Gly pretreatment at 150 °C for 15 h (Hossain et al. 2021), while Xu et al. (2018) reported a much lower delignification efficiency of ≈10% from corn stover after ChCl:Gly (1:2) pretreatment at 160 °C for few hours. Other researchers reported delignification efficiency of 20.8% from corncobs after ChCl:Gly pretreatment at 150 °C for 15 h (Procentese et al. 2015). Furthermore, Chen et al. (2018) reported ≈8% of delignification from switchgrass pretreated with ChCl:Gly at 120 °C for 1 h. Other researchers reported a delignification for 49% from oil palm trunk pretreated with ChCl:Gly at 100 °C for 48 h (Zulkefli et al. 2017).

### Effect on composition

Figure 1g–i shows the effects of DES pretreatment on the composition of rice straw. The untreated sample consists of cellulose (41.8%), hemicellulose (24.9%), lignin (17.0%), ash (15.0%) and others (1.3%). Hou et al. (2018) reported 35.5% cellulose, 20.6% xylan and 21.5% lignin from untreated rice straw. As shown in Fig. 1g, cellulose content increased from 52.8% (100 °C) to 57.6% (140 °C) after ChCl:Gly pretreatment, while after ChCl:FA pretreatment at 100 °C and 140 °C, cellulose content of 48.5% and 58.2%, respectively were obtained. On the contrary, hemicellulose and lignin contents decreased with increases in temperature after both ChCl:Gly and ChCl:FA pretreatment. ChCl:Gly and ChCl:FA pretreatment at 140 °C resulted in hemicellulose content of 19.3% and 11.1%, respectively. Similarly, lignin content decreased with temperature increases reaching 8.2% and 9.8% after ChCl:Gly and ChCl:FA pretreatment at 140 °C, respectively. The ash content increased after ChCl:FA pretreatment reaching 19.9% at 140 °C, while ash content decreased to 12.7% after ChCl:Gly pretreatment at 120 °C. Others ranged from 1.1 to 1.9% after ChCl:Gly and ChCl:FA pretreatment.

In Fig. 1h, cellulose content increased with temperature, reaching 59.4% and 58.1% after ChCl:Gly and ChCl:FA pretreatment at 140 °C, respectively. However, hemicellulose content decreased with temperature increases, reaching 17.2% at 140 °C after ChCl:Gly pretreatment compared to 10.9% after ChCl:FA pretreatment at the same temperature. The lignin content decreased with temperature increases, reaching 8.1% and 9.6% after ChCl:Gly and ChCl:FA pretreatment at 140 °C, respectively. The ash content increased from 12.4% to 13.9% after ChCl:Gly pretreatment with increases in temperature. In contrast, the ash content increased after ChCl:FA pretreatment at 140 °C, reaching 20.4% compared to 14.0% after ChCl:Gly pretreatment at the same temperature. Others increased slightly from 1.0% to 1.3% after pretreatment with increases in temperature increases.

As shown in Fig. 1i, temperature increases resulted in increased cellulose content in both ChCl:Gly and ChCl:FA pretreatment, reaching 59.8% and 59.1% at 140 °C, respectively. On the contrary, hemicellulose content decreased with temperature increases, reaching 15.7% and 10.1% after ChCl:Gly and ChCl:FA pretreatment, respectively. Similarly, temperature increases decreased lignin content, reaching 8.2% after ChCl:Gly pretreatment at 140 °C compared to 9.6% of lignin content after ChCl:FA pretreatment at the same temperature. After ChCl:Gly pretreatment at 140 °C, ash content of 15.0% was obtained, compared to 20.2% after ChCl:FA pretreatment at 140 °C. Others had no appreciable change after ChCl:Gly and ChCl:FA pretreatment.

Cellulose content correlated with temperature as temperature increases resulted in cellulose enrichment, while increases in reaction time showed minimal impact on cellulose enrichment. This apparent lack of correlation between reaction time and cellulose enrichment could indicate lignin depolymerization when reaction time becomes prolonged. Other authors reported cellulose and xylan content of 35.9% and 19.7% from rice straw after ChCl:Gly pretreatment at 120 °C for 6 h without microwave irradiation treatment (Hou et al. 2018). On the other hand, higher cellulose content of around 48% was from rice hull by combining ChCl:Gly pretreatment and microwave irradiation treatment at 155 °C for 5 min (Kumar et al. 2019). Other authors reported 58.4% of glucan content from rice straw pretreated with ChCl:pTSA:EG under microwave conditions of 98 °C and 33 min (Poy et al. 2023). Isci et al. (2020) reported 55.6% and 7.3% of glucan and xylan content from wheat straw after ChCl:FA pretreatment under microwave conditions of 450 W and 8 min.

The relatively high lignin content after ChCl:FA pretreatment compared to ChCl:Gly in the present study may be connected to repolymerization of lignin at high temperatures (≥ 130 °C) as lignin tends to recondense into the solid residue (Kohli et al. 2020). However, the lignin content of 9.6% after ChCl:FA at 140 °C in the present study was lower than the 10.9% reported by other researchers from a softwood mixture pretreated with ChCl:FA at 160 °C (Ceaser et al. 2023). Other authors reported a much lower lignin content of 5.8% after ChCl:AA pretreatment at 140 °C for 105 min was applied to rice straw (Maibam et al. 2022).

### Effect on cellulose crystallinity, functional groups and surface morphology

**Fig. 2:**
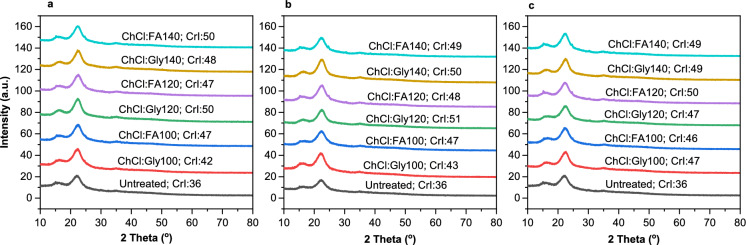
Effect of ChCl:Gly and ChCl:FA pretreatment on crystallinity: **a** Pretreatment at 100–140 °C for 5 min; **b** Pretreatment at 100–140 °C for 10 min;** c** Pretreatment at 100–140 °C for 15 min

X-ray diffraction (XRD) is a valuable tool for determining the effect of pretreatment on the crystal structure of rice straw biomass. The crystallinity index (CrI) is a quantitative analysis used to assess the crystalline material in cellulose (Salem et al. 2023). Because of its linear (unbranched) structure made of a single monosaccharide, cellulose is more crystalline than hemicellulose or lignin (Cheng et al. 2022; Kondo 1997). As seen from XRD spectra (Fig. 2), there were characteristic peaks at around 16.0° and 22.5° for the untreated and pretreated rice straw. These peaks are typical for the cellulose Iβ crystal form, similar to simulated patterns for cellulose (Nam et al. 2016).

In Fig. 2a, CrI obtained after ChCl:Gly pretreatment ranged from 42 to 50%, and 47–50% after ChCl:FA pretreatment compared to CrI of 36% obtained for the untreated. Further increases in residence time resulted in CrI ranging from 43 to 51% after ChCl:Gly pretreatment and 47–49% after ChCl:FA pretreatment (Fig. 2b). In Fig. 2c, the CrI after ChCl:Gly pretreatment ranged from 47 to 49% and 46–50% after ChCl:FA pretreatment. ChCl:Gly pretreatment at 120 °C, 10 min resulted in the highest CrI of 51%, while the lowest CrI of 43% resulted from ChCl:Gly pretreatment at 100 °C, 5 min. Increases in residence from 5 to 15 min during pretreatment at higher temperatures appeared not to affect the CrI significantly.

However, ChCl:Gly and ChCl:FA pretreatment displayed similarities in CrIs due to the close resemblance of their cellulose content. This high CrIs indicates that pretreatment caused a partial disruption and loss of the amorphous regions of the cellulose, thereby exposing the highly crystalline cellulose with high CrI (Liang et al. 2020). Since CrI is directly related to the amount of crystalline cellulose present in a sample, thus the high CrI obtained after ChCl:Gly and ChCl:FA pretreatment may be due to cellulose enrichment. Sawhney et al. (2023) reported a CrI of 57% for untreated rice straw and 61% following microwave-assisted ChCl-FA pretreatment, indicating a 4% increase compared to ≈15% increase in CrI obtained in the present study following ChCl:Gly and ChCl:FA pretreatment. These results demonstrate the efficiency of ChCl:Gly and ChCl:FA pretreatment on delignification and cellulose enrichment. Thus, the hydrogen bonds between the cellulose molecules are in the ordered system (Sánchez et al. 2016). ChCl:Gly and ChCl:FA pretreatment results in the removal of the amorphous portion of cellulose, hemicellulose, and lignin due to damage caused to the fibre structure; hence, more of the cellulosic surface becomes exposed (Roy et al. 2020).

**Fig. 3:**
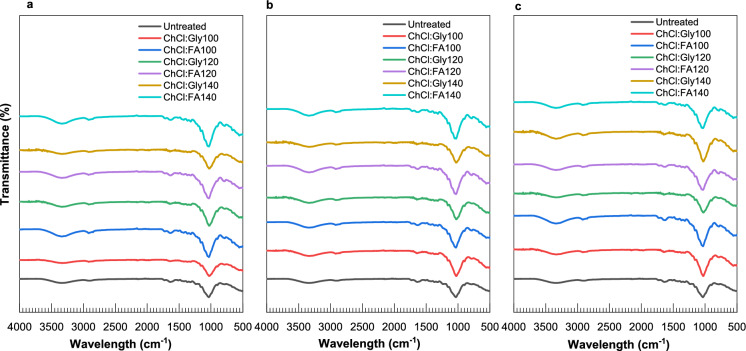
Effect of ChCl:Gly and ChCl:FA pretreatment on the functional groups: **a** Pretreatment at 100–140 °C for 5 min; **b** Pretreatment at 100–140 °C for 10 min; **c** Pretreatment at 100–140 °C for 15 min

FTIR analysis provided insights into the effects of pretreatment on the chemical groups in lignocellulose. Peaks were observed at 3345 cm^−1^, 2906 cm^−1^, 1654 cm^−1^ and 1039 cm^−1^, respectively, in all the samples and peak intensity varied as shown in (Fig. 3a–c). The peaks at 3345 cm^−1^ (O–H stretching vibration) were broader in all pretreated samples compared to the untreated due to increases in cellulose content. In this region, peak intensities decreased after ChCl:Gly pretreatment, while peak intensity increased after ChCl:FA pretreatment, indicating lower hemicellulose content after ChCl:FA pretreatment. ChCl:FA pretreatment resulted in hemicellulose degradation due to the strong interactions between its acidic components and the hemicellulose structure (Zhang et al. 2025a, b). The decreased peak intensities obtained at 2906 cm^−1^ (CH_2_ and C–H vibrations) after pretreatment indicate the presence of cellulose and hemicellulose.

On the other hand, a peak at 1654 cm^−1^ indicates a functional group of carbonyl (C=O) stretching from hemicellulose. At this region, peak intensities increased after ChCl:FA pretreatment, while peak intensity decreased after ChCl:Gly pretreatment, indicating low hemicellulose content after ChCl:FA pretreatment. The peak at 1039 cm^−1^ is for C–O from guaiacyl ring in lignin (Zulkefli et al. 2017). The peak intensities in this region strongly decreased after ChCl:Gly and ChCl:FA pretreatment, indicating that pretreatment significantly removed lignin. FTIR results correlated with the composition analysis, suggesting that FTIR could provide quantitative data to understand better the effect of pretreatment on the chemical structure of biomass.

**Fig. 4:**
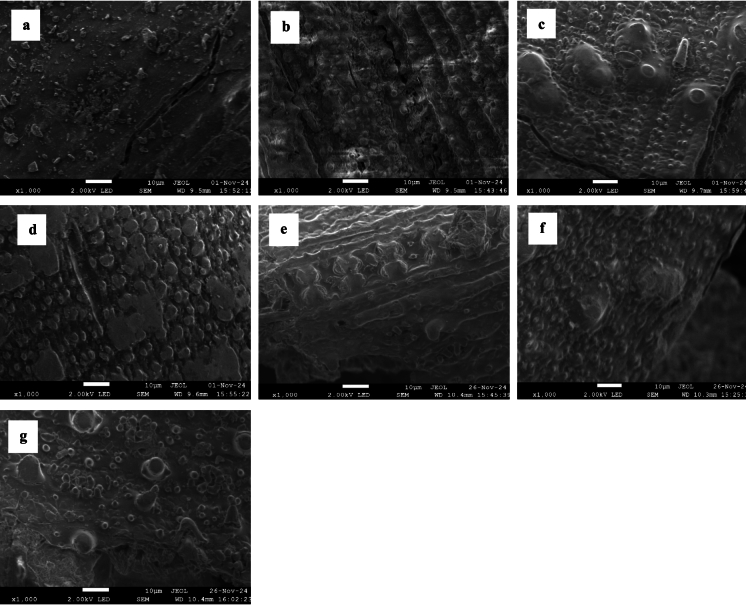
SEM images: **a** Untreated; **b** ChCl:Gly pretreatment at 100 °C for 15 min; **c** ChCl:Gly pretreatment at 120 °C for 10 min; **d** ChCl:Gly pretreatment at 140 °C for 10 min; **e** ChCl:FA pretreatment at 100 oC for 10 min;** f** ChCl:FA pretreatment at 120 oC for 15 min; **g** ChCl:FA pretreatment at 140 oC for 10 min; All images were taken at 10 µm and 1000 × magnification

Figure 4a–g shows the SEM images of untreated and pretreated samples. SEM analysis allows the accessible surface area of LCB to be assessed and can be a valuable tool for predicting enzymatic digestibility (Zoghlami and Paës 2019). The untreated rice straw exhibited a relatively smooth surface compared to the pretreated samples (Fig. 4a). All the pretreated samples showed a typical rough surface with pores, and these pores appeared to get larger as the temperature and residence time increased in the ChCl:Gly pretreated samples (Fig. 4c, d). Similarly, large pores were present in ChCl:FA pretreated samples at higher temperatures (Fig. 4f, g). Thus, the large pores seen after pretreatment at higher temperatures indicate the breaking of chemical bonds between lignin and carbohydrates (Giummarella and Lawoko 2016; Zhao et al. 2020). However, there appears to be a strong correlation between pore size on the surface of biomass and enzymatic digestibility (Peciulyte et al. 2015). Therefore, pretreated samples with larger pores on their surfaces may improve enzymatic digestibility compared to smaller pores.

### Effect on thermal degradation

**Fig. 5:**
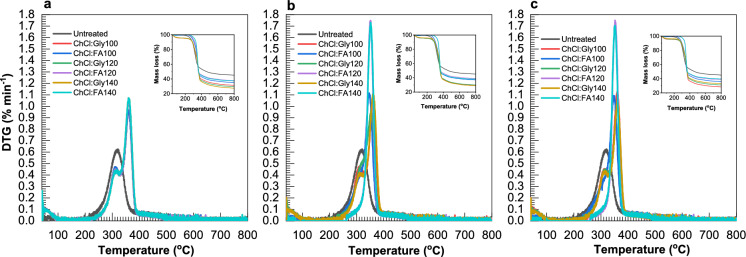
Effect of ChCl:Gly and ChCl:FA pretreatment on thermal degradation: **a** Pretreatment at 100–140 °C for 5 min; **b** Pretreatment at 100–140 °C for 10 min; **c** Pretreatment at 100–140 °C for 15 min

The chemical components of lignocellulosic biomass show differences in thermal stability with cellulose and hemicellulose decomposing within a narrow temperature range mainly due to glycosidic linkages (Steinbach et al. 2019). However, the more amorphous hemicellulose decomposes at lower temperature than the crystalline cellulose, whereas lignin degradation occurs over a broader temperature range (Chen et al. 2012). Figure 5 shows the effect of pretreatment on the thermal degradation of rice straw. Pretreatment resulted in significant increases in mass loss compared to untreated. This increases in mass loss indicate that pretreatment disrupts the biomass structure, resulting in lignin solubilization and removal. Lower mass losses were observed in samples with prolonged pretreatment time, indicating the presence of more crystalline cellulose in these samples.

Two distinct degradation peaks occurred at 320 °C and 360 °C for ChCl:Gly and ChCl:FA pretreated samples, corresponding to degradation peaks for hemicellulose and cellulose, respectively. This observation is consistent with the study conducted by Mao et al. (2025) using Oak and pine wood chips and PTSA:ChCl:Glycerol (2:1:1). However, a broad peak occurred in the untreated at 320 °C indicating that cellulose and hemicellulose degradation occurred within the same temperature range. In Fig. 5a, shoulder peaks 320 °C were observed in all ChCl:Gly pretreated despite increases in time, while these peaks disappeared in ChCl:FA pretreated samples over a longer time (10–15 min). The decomposition rate observed for samples pretreated at 5 min was similar for both ChCl:Gly and ChCl:FA pretreatment. When time becomes prolonged during pretreatment, a higher decomposition rate (1.7% min^−1^) occurred at 360 °C after ChCl:FA pretreatment (Fig. 5b, c).

On the other hand, there was no change in decomposition rate after ChCl:Gly pretreatment. The decomposition rate remained unchanged at around 1.0% min^−1^. This difference indicates that increases in time during ChCl:Gly pretreatment did not significantly alter the biomass structure compared to ChCl:FA pretreatment, where increases in time resulted in significant changes in the biomass structure. DES pretreatment removed lignin, thereby exposing hemicellulose and cellulose to thermal decomposition. A higher decomposition rate occurred at 360 °C in ChCl:FA pretreated samples, indicating that ChCl:FA pretreatment reduced cellulose crystallinity compared to ChCl:Gly pretreatment.

### Effect on enzymatic hydrolysis

**Fig. 6:**
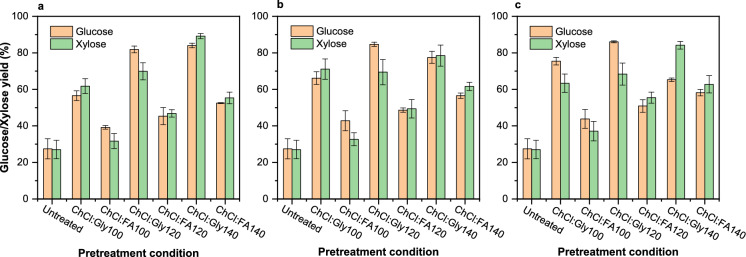
Effect of ChCl:Gly and ChCl:FA pretreatment on glucose and xylose yield after 72 h of enzymatic hydrolysis; Average values are presented, and the error bars correspond to standard deviation

Figure 6 shows the effect of DES pretreatment on fermentable sugar yield after 72 h of enzymatic hydrolysis. Glucose and xylose yields of 27.5% and 27.1%, respectively, were obtained from the untreated sample. In Fig. 6a, increases in pretreatment temperature during pretreatment resulted in corresponding increases in the yield of glucose in both ChCl:Gly and ChCl:FA pretreated samples, but ChCl:Gly pretreatment resulted in higher increases in glucose yield (56.5–84.0%) compared to glucose yield of 39.2–52.5% after ChCl:FA pretreatment. Similarly, ChCl:Gly pretreatment at 140 °C yielded a xylose yield of 89.1% compared to 69.9% after ChCl:FA pretreatment at 120 °C. In Fig. 6b, temperature increases resulted in a corresponding increase in glucose and xylose yield after ChCl:Gly and ChCl:FA pretreatment. ChCl:Gly pretreatment at 120 °C yielded 84.7% of glucose compared to 56.5% of glucose yield after ChCl:FA pretreatment at 140 °C. ChCl:Gly pretreatment at 140 °C resulted in a xylose yield of 78.5% compared to a xylose yield of 61.6% after ChCl:FA pretreatment at the same temperature.

As shown in Fig. 6c, temperature increases resulted in corresponding increases in glucose yield, reaching 86.0% after ChCl:Gly pretreatment at 120 °C and decreasing to 65.3% after ChCl:Gly pretreatment at 140 °C. Similarly, glucose yield increased with temperature, reaching 58.2% after ChCl:FA pretreatment at 140 °C from 43.8% after ChCl:FA pretreatment at 100 °C. Xylose yield increased to 84.1% and 62.8% after ChCl:Gly and ChCl:FA pretreatment at 140 °C. The decreases in glucose yield after pretreatment at 140 °C suggest that the high ash content might have hindered enzyme access to cellulose. The low yield of glucose and xylose after ChCl:FA pretreatment compared to ChCl:Gly pretreatment may be due to the high ash content after ChCl:FA pretreatment. A similar decrease in glucose yield resulted after ChCl:Gly pretreatment at 140 °C, when the ash content reached 15%. Other authors reported that the glucose content of sulfuric acid pretreated corn stover increased from 43.30 to 70.99% after a washing step with tap water reduced the ash content from 9.60 to 4.98% (He et al. 2014). Other studies show that the impact of high ash content on enzymatic hydrolysis increases mainly following acid pretreatment (Fitria et al. 2025; Yang et al. 2018). Thus, acidic conditions during biomass pretreatment could decrease enzymatic digestibility.

On the other hand, the effect of lignin content on enzymatic digestibility obtained in this study was consistent with the reports from different authors in which lignin content above 10% significantly reduced cellulose conversion into glucose (Igbojionu et al. 2020; Zhang et al. 2016). This decrease is mainly due to the nonproductive binding of lignin to cellulase, thereby physically blocking enzyme access to cellulose (Qing and Wyman 2011). Furthermore, the crystallinity of cellulose affects enzymatic hydrolysis as previously reported in the literature (Ling et al. 2017). However, in the present study, there was no correlation between cellulose crystallinity and glucose yield specifically after ChCl:Gly pretreatment, suggesting the presence of more amorphous than crystalline cellulose, whereas the low glucose and xylose yields after ChCl:FA pretreatment may be due to the presence of highly crystalline cellulose. The results of TGA and DTG confirm the presence of crystalline cellulose after ChCl:Gly pretreatment compared to relatively more amorphous cellulose after ChCl:FA pretreatment. In addition, changes in biomass surface morphology (pore sizes) correlated with sugar yield mainly after ChCl:Gly pretreatment, as high glucose and xylose yields resulted from samples with large pores on their surfaces. On the contrary, cellulose digestibility was poor despite the large pores on the fibre surface after ChCl:FA pretreatment, indicating cellulose inaccessibility to cellulase due to high ash content.

## Conclusion

ChCl:Gly pretreatment achieved a higher delignification (54.4%) compared to ChCl:FA pretreatment (40.4%), although their cellulose contents were closely related (59.8% and 59.1%). However, ChCl:FA pretreatment led to significant hemicellulose degradation (60%) compared to ChCl:Gly pretreatment (36%). ChCl:FA pretreatment led to high ash (20.2%) generation, compared to ChCl:Gly pretreatment with maximal ash content of 15.0%. Thus, the high ash content could be a drawback with ChCl:FA pretreatment under microwave conditions, especially for ash-rich LCBs such as rice straw.

Even though lignin removal with DESs (~ 54.4%) might be lower than some organic solvents, DES pretreatment offers a greener, safer, cost-effective, and customizable alternative—providing a strong balance of performance with environmental and operational benefits. ChCl:Gly pretreatment improved cellulose digestibility with a high glucose yield (86.0%) compared to 58.2% after ChCl:FA pretreatment and glucose yield after ChCl:Gly pretreatment was over three-fold higher than the untreated (27.5%). Thus, in the biorefinery concept requiring a high yield of fermentable sugar for bioethanol production, ChCl:Gly pretreatment will be preferable to ChCl:FA pretreatment under microwave conditions. One drawback with ChCl:Gly pretreatment is that three washing steps are required to remove the residual glycerol, leading to high water usage. Additionally, scaling up microwave-assisted DES pretreatment for industrial applications presents key challenges. Therefore, substantial research efforts towards reactor design, process optimization, and solvent management are key to allowing practical and economically viable industrial deployment.

## Data Availability

No datasets were generated or analysed during the current study.
